# Gunshot injury to the colon by expanding bullets in combat patients wounded in hybrid period of the Russian-Ukrainian war during 2014–2020

**DOI:** 10.1186/s12893-023-01919-6

**Published:** 2023-01-27

**Authors:** Kostiantyn Gumeniuk, Igor A. Lurin, Ievgen Tsema, Lesia Malynovska, Maksym Gorobeiko, Andrii Dinets

**Affiliations:** 1 Medical Forces Command, Armed Forces of Ukraine, Kyiv, Ukraine; 2 Department of Military Surgery, Ukrainian Military Medical Academy, Kyiv, Ukraine; 3 National Academy of Medical Sciences of Ukraine, Kyiv, Ukraine; 4 State Institution of Science “Research and Practical Center of Preventive and Clinical Medicine”, State Administrative Department, Kyiv, Ukraine; 5 Department of Surgery, Bogomolets National Medical University, Kyiv, Ukraine; 6 Department of Surgery, Institute of Biology and Medicine, Taras Shevchenko National University of Kyiv, Demiїvska 13, Kyiv, 03039 Ukraine

**Keywords:** Colon injury, Gunshot, War in Ukraine, Shape-stable bullets, Hollow-point bullet, Expanding bullet

## Abstract

**Background:**

A gunshot wound to the colon is a frequent injury in armed conflicts. An example of a high-energy modern weapon is hollow-point bullets, which is associated with increased tissue damage and lethal outcome. The aim of this study was to evaluate gunshot injuries to the colon in combat patients and to assess the difference in clinical features of patients with colon injuries by hollow-point versus shape-stable bullets.

**Patients and methods:**

Analyses of clinical data were performed on 374 male soldiers from the Armed Forces of Ukraine with gunshot abdominal wounds with injury to the colon in East Ukraine between 2014 and 2020. Out of 374 injured, 112 (29.9%) patients were diagnosed with penetrating gunshot bullet wounds: 69/112 (61.6%) were injured by shape-stable bullets, and the hollow-point bullets injured 43/112 (38.4%) patients.

**Results:**

More severe hemorrhagic shock stages were in patients injured by hollow-point bullets: shock stages III-IV was in 25 (58.1%) patients injured by the hollow-point bullets *vs.* 17 (24.6%) patients injured by shape-stable bullets (p = 0.0004). Left colon parts were more frequently injured as compared to the right colon side or transverse colon: 21 (48.8%) patients were injured by the hollow-point bullets (p < 0.0001), and 41 (59.4%) patients were injured by the shape-stable bullets (p = 0.032). A significant difference was identified for the frequent injury to the middle colon within the entire cohort (p = 0.023). Patients injured by the hollow-point bullets demonstrated a higher frequency of 3–5 areas of colon gunshot defects, which was detected in 18 (41.8%) patients injured by hollow-point bullets and none with shape-stable bullets injury (p = 0.0001). Colon Injury Scale (CIS) IV was detected in 7 (16.3%) patients injured by the hollow-point bullets as compared to 2 (2.9%) patients injured by shape-stable bullets (p = 0.011). Colostomy was performed in 14 (69%) patients injured by shape-stable bullets and in 12 (27.9%) patients injured by hollow-point bullets (p > 0.05). 15 (35%) patients died after injury by the hollow-point bullet, whereas 9 (13%) patients after damage by the shape-stable bullets (p = 0.0089).

**Conclusions:**

All patients should be suspected to have an injury by bullet with expanding properties in case of penetrating abdominal injury (absent of outlet wound) and careful revision of the abdomen must be performed to identify possible multiorgan injury as well as multiple gunshot defects of the intestine.

**Supplementary Information:**

The online version contains supplementary material available at 10.1186/s12893-023-01919-6.

## Introduction

A gunshot wound to the colon is a frequent injury in armed conflicts, which is associated with severe trauma in combatants due to the increased damage properties of modern high-energy weapons [1, 2]. Such gunshot injuries may cause massive damage to soft tissue and internal organs, resulting in lethal outcomes or more prolonged treatment and rehabilitation time [3, 4]. The Russian-Ukrainian war was started in 2014, and for the first eight years, this war was ongoing as hybrid warfare, followed by the initiation of direct fights between the two armies in 2022 [5, 6]. During the hybrid period of 2014–2020, and nowadays, we have noticed Russian army frequently uses various types of weapons with high damage properties (*e.g.* hollow-point bullets), which is prohibited by international law [7]. Considering this fact, military medical officials in Ukraine came to the question of whether or not the abovementioned types of bullets impact the severity of gunshot wounds and specific locations of the injuries, as well as relations to the survival of the patients and their treatment. The results were used to update the clinical guidelines and upgrade military hospitals at the appropriate Level to be ready to provide aid in case of prohibited high-energy weapons, including hollow-point bullets. In this study, we have focused on analyses of colon injury in the abovementioned conditions. Military medical doctors of Ukraine were challenged with the management and rehabilitation of military personnel who were injured by modern high-energy weapons and received severe abdominal trauma. An example of such a modern weapon is a hollow-point bullet, which is a specially shaped bullet expanding into multiple smaller projectiles upon impacting the target and causing severe injury to soft tissues and internal organs. The hollow-point bullet could perforate high-class body armor [8, 9]. Such ballistic properties might be associated with severe abdominal trauma: multiorgan injury, multiple perforations of the bowel with massive fecal diversion, as well as a high risk of bleeding with hemorrhagic shock and retroperitoneal hematomas [10, 11]. Hollow-point bullet ballistic characteristics are also associated with multiple full-thickness gunshot defects to the colon wall, whereas single defect of the colon wall is uncommon [8, 12].

Such gunshot trauma with hollow-point bullets could be associated with a lethal outcome to combat patients, even concerning the principle of “golden hour” and damage control surgery. Ballistic features of the hollow-point bullets envisage expanding on impact followed by the distribution of multiple fragments or bullet mushrooming, causing more damage to tissues. Using such features, the X-ray became an effective tool for detecting metal fragments in the abdomen and establishing the mechanism of injury. Little is known about the gunshot injury to the colon in the warfare in Ukraine. The aim of this study was to evaluate gunshot injuries to the colon in combat patients and to assess the difference in clinical features of patients with colon injuries by hollow-point versus shape-stable bullets.

## Materials and patients

All soldiers from the Armed Forces of Ukraine with gunshot abdominal trauma and injury to the colon in East Ukraine between 2014 and 2020 were eligible for this retrospective study. In this study, we hypothesize that patients with gunshot injuries to the colon by hollow-point bullets have different clinical features and outcomes compared to those injured by shape-stable bullets. Evaluation of the patients was performed concerning the bullet types and depended on the presence or absence of fragmentation hollow-point bullets *vs.* shape-stable bullets. Patients were not included in the study in case of injury by explosive munitions (artillery, land mines, grenades, etc.). There were identified 374 male patients who were surgically treated at a military field hospital (Level II of medical care) situated in a 20 km zone from the frontline. The term “Level” is used in the National Military Doctrine of Ukraine, aiming to show the stratification of the five tiers in which medical aid is provided [3, 6, 7]. Each Level is a kind of medical tier to be organized on a progressive basis from 1 to 5 to apply treatment, evacuation, specific approaches to patient support as well as rehabilitation. Level I is related to First aid, which is organized during the evacuation or at the point of injury. Level II is corresponded to the military field hospital with a limited inpatient bed space, functioning within a distance of 20–40 km from the frontline. Laparoscopy is available in hospitals of Level II. Level III is specialized medical and surgical care, which is provided in a large hospital with a bulk of inpatients beds, within a distance of over 40 km from the frontline, in cities like Kharkiv, Dnipro, Odesa, Vinnytsya, Kyiv, where advanced equipment (CT, MRI, etc.) and high-skilled surgeons are available. Level IV is a tier of highly specialized medical aid outside of the battlefield zone, providing an application of high-tech medical equipment for patients who require more prolonged treatment or who need medical capabilities found inadequate at lower Level II/III. Level V is a tier of rehabilitation facilities.

Out of 374 patients with a gunshot injury to the colon, 112 (29.9%) patients with penetrating bullet wounds comprised the study group, whereas 262 (70.1%) patients were not included in the because of penetrating gunshot wounds due to shrapnel injury. Out of 112 patients from the study group, 69 (61.6%) were injured by shape-stable bullets, and hollow-point bullets injured 43 (38.4%) patients. Evacuation of injured patients from the battlefield was performed within the “golden hour” [13].

All patients received medical aid at Level II. At admission to Level II hospital, all patients were diagnosed with gunshot injuries and hemorrhagic shock requiring resuscitation at the intensive care unit (ICU). Shock severity was determined according to the American College of Surgeons Advanced Trauma Life Support (ATLS). Damage control tactics was applied to all patients. Injury to the colon was evaluated according to the Colon Injury Scale (CIS).

Computed tomography was not available at Level II. Abdominal X-ray was applied to detect the localization of the projectile or its fragments orientation (tumbling), deformation, and fragmentation (number of fragments and area of fragments dissemination).

Right colon parts constituted cecum, ascending colon, and hepatic flexure. Left colon parts formed splenic colon flexure, descending, and sigmoid colon. As upper colon, we considered the transverse colon, hepatic and splenic flexures, middle colon—ascending and descending colon, and lower colon—cecum and sigmoid colon.

### Statistical analyses

Statistical analyses were performed using the software SPSS v.22 (IBM, USA). A chi-squared test (χ^2^-test) was applied to test the null hypothesis for categorical values, whereas Student’s t-test and One-way analysis of variance were used for continuous variables. Statistical difference was considered as significant at p < 0.05.

## Results

The mean age of all patients was 24.7 years (range 19–47 years). 82 (73.2%) patients were evacuated within the 1 h (mean time 55.3 ± 1.17 min) from the moment of injury until the admission to Level II, which is in line with the principle of “golden hour.”

Data analyses are presented in the Tables. Detailed statistical data are shown in Additional file 1: Table S1, Additional file 2: Table S2, Additional file 3: Table S3. Analyses of hemorrhagic shock stages are presented in Table 1 and Additional file 1: Table S1. A hemorrhagic shock Stage III was diagnosed in 18 (41.9%) patients injured by the hollow-point bullets, which is more frequent as compared to 13 (18.8%) patients injured by shape-stable bullets (p = 0.0081). In contrast, hemorrhagic shock Stage I was diagnosed in 2 (4.7%) patients injured by the hollow-point bullets, which were less frequent as compared to 25 (36.2%) patients injured by shape-stable bullets (p = 0.0001). Shock Stages I-II were detected in 52 (75.4%) patients injured by shape-stable bullets as compared to 18 (41.9%) patients injured by hollow-point bullets (p = 0.0004).

**Table 1:** Analyses of the hemorrhagic shock severity in relation to cause of gunshot injury by shape-stable or hollow-point bullets

Shock severity		Grouping of patients		All patients n = 112	p value
		Injured by Shape-stable bullets n = 69	Injured by Hollow-point bullets n = 43		
Stages	I	25 (36.2%)	2 (4.7%)	27 (24.1%)	0.0001
	II	27 (39.1%)	16 (37.2%)	43 (38.4%)	0.8389
	III	13 (18.8%)	18 (41.9%)	31 (27.7%)	0.0081
	IV	4 (5.8%)	7 (16.3%)	11 (9.8%)	0.0699

**Fig. 1:**
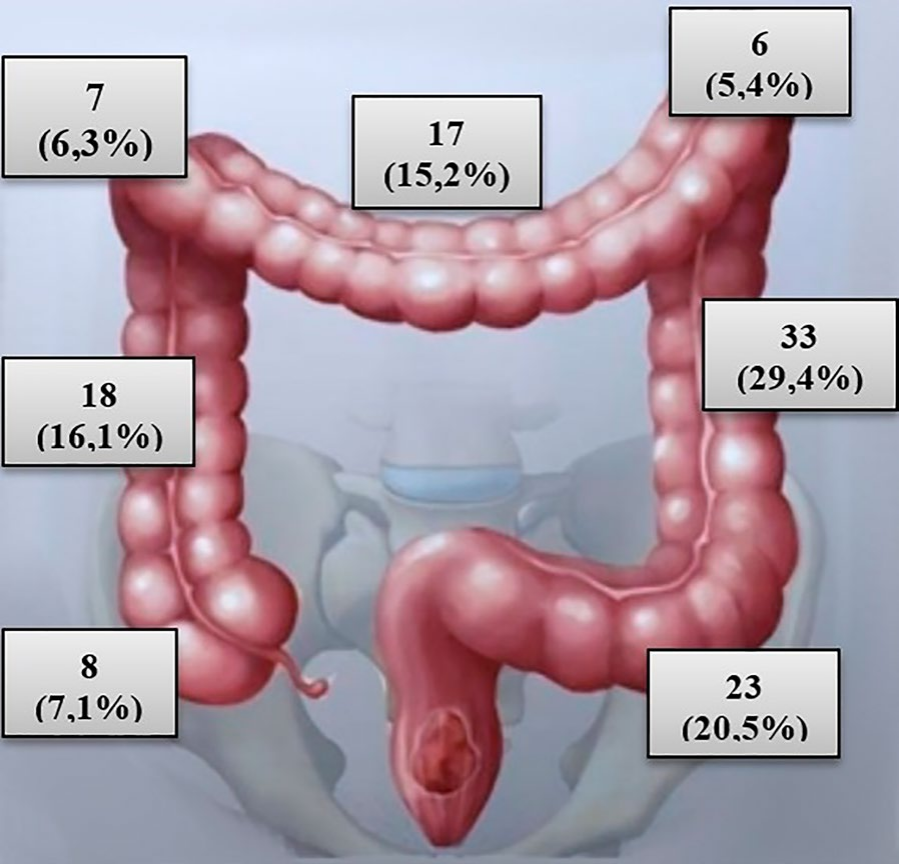
Schematic illustration of gunshot injury to the various parts of the colon of study cohort

**Fig. 2:**
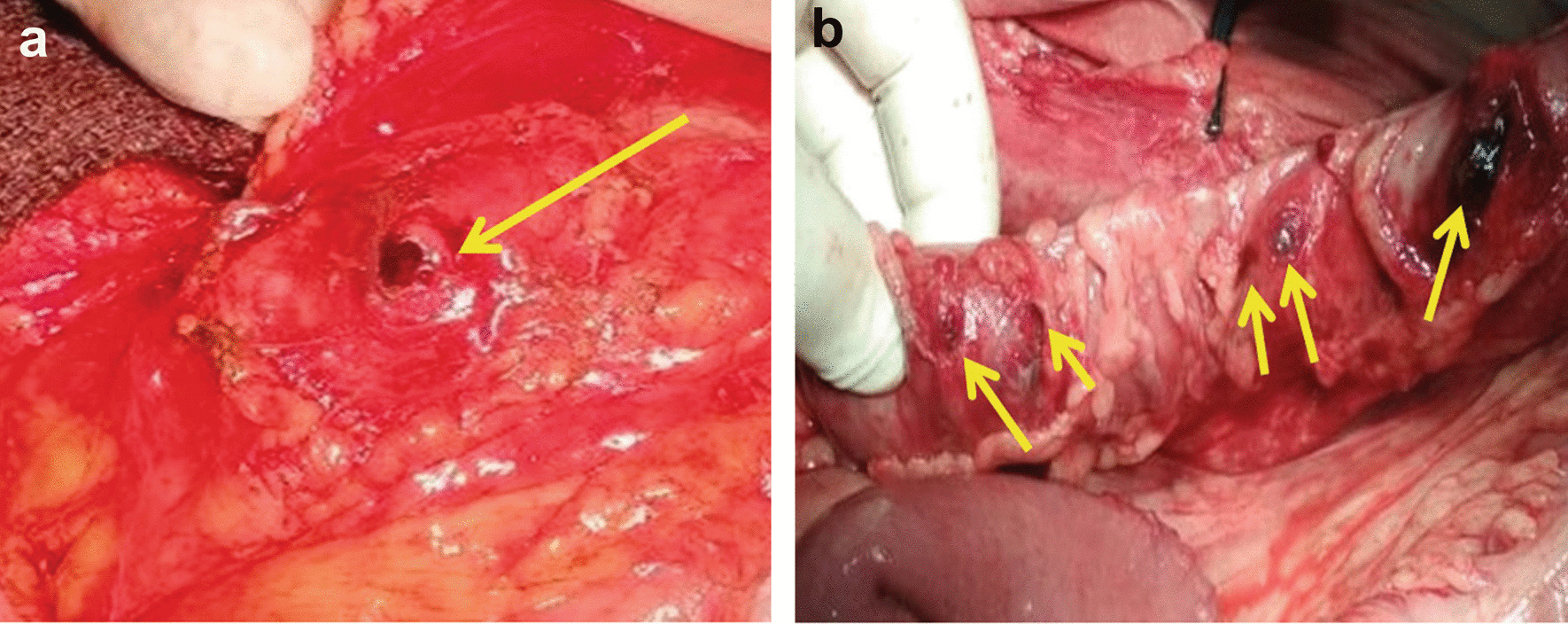
Intraoperative photograph of colon gunshot injury. **A** A single wound to the colon (entry gunshot hole marked with an arrow). **B** Multiple injuries to the colon by fragments of bullet (marked with arrows)

At laparotomy or laparoscopy, all 112 (100%) patients were diagnosed with various localization of damages to the colon wall (Table 2, Figs. 1, 2). Data analyses showed that left colon parts were more frequently injured as compared to right colon or transverse colon in 21 (48.8%) injured by the hollow-point bullets (p < 0.0001) and in 41 (59.4%) patients injured by the shape-stable bullets. Data analyses showed that 80 (71.4%) patients were diagnosed with a gunshot injury to the left side and middle colon.

**Table 2:** Analyses of colon injury by localizations of gunshot defects by shape-stable or hollow-point bullets

Colon parameters	Grouping of patients		All patients n = 112	p value
	Injured by Shape-stable bullets n = 69	Injured by Hollow-point bullets n = 43		
Colon part				
Cecum	6 (8.7%)	2 (4.7%)		
Ascending colon	9 (13.0%)	9 (20.9%)	18 (16.1%)	0.27
Hepatic flexure	3 (4.3%)	4 (9.3%)	7 (6.3%)	0.29
Transverse colon	10 (14.5%)	7 (16.3%)	17 (15.2%)	0.80
Splenic flexure	5 (7.2%)	1 (2.3%)	6 (5.4%)	0.26
Descending colon	21 (30.4%)	12 (27.9%)	33 (29.5%)	0.78
Sigmoid colon	15 (21.7%)	8 (18.6%)	23 (20.5%)	0.69
p-value—comparisons within each group	0.0005	0.0171	< 0.0001	
Colon side				
Right-side colon	18 (26.1%)	15 (34.9%)	33 (29.5%)	0.32
Transverse colon	10 (14.5%)	7 (16.3%)	17 (15.2%)	0.80
Left-side colon	41 (59.4%)	21 (48.8%)	62 (55.4%)	0.27
p-value—comparisons within each group	< 0.0001	0.032	< 0.0001	
Colon level				
Upper colon	18 (26.1%)	12 (27.9%)	30 (26.8%)	0.83
Middle colon	30 (43.5%)	21 (48.8%)	51 (45.5%)	0.58
Lower colon	21 (30.4%)	10 (23.3%)	31 (27.7%)	0.41
p-value—comparisons within each group	0.18	0.091	0.023	
Relation to peritoneum				
Intraperitoneal	39 (56.5%)	22 (51.2%)	61 (54.5%)	0.58
Extraperitoneal	30 (43.5%)	21 (48.8%)	51 (45.5%)	
p-value—comparisons within each group	0.44	0.91	0.50

Analyses of the entire cohort showed multiple injuries to the colon in 65 (58%) patients, whereas a single colon gunshot wound was detected in 47 (42%) patients (Table 3). All 43 (100%) patients injured by hollow-point bullet injury had multiple wounds to the colon (2 or more defects), which is 3 times more frequent as compared to 22 (31.8%) patients injured by shape-stable bullets with borderline significance (p = 0.0488). Also, 18 (41.8%) patients injured by the hollow-point bullets demonstrated a higher frequency of 3–5 defects of the colon wall, compared to the absence of the patients with shape-stable bullets injury (p = 0.0001). Wounds to the colon with ≥ 5 defects of the colon wall were identified in 4 (9.3%) patients injured by the hollow-point bullets, but none of the patients with shape-stable bullets injury (p = 0.0199).

**Table 3:** Analyses of colon injury by localizations and number of gunshot defects by shape-stable or hollow-point bullets

Colon injury location	Grouping of patients					
	Injured by Shape-stable bullets n = 69			Injured by Hollow-point bullets n = 43		
Number of colon defects, n =	2	3–4	≥ 5	2	3–4	≥ 5
Cecum, n =	0	0	0	1	1	0
Ascending colon, n =	3	0	0	5	4	0
Hepatic flexure, n =	2	0	0	3	1	0
Transverse colon, n =	1	0	0	2	4	1
Splenic flexure, n =	3	0	0	0	1	0
Descending colon, n =	4	0	0	7	3	2
Sigmoid colon, n =	9	0	0	3	4	1
Total	22* (31.8%)	0	0	21 (48.8%)	18 (41.8%)**	4 (9.3%)***

p-value: *0.0488, **0.0001, ***0.0199

CIS analyses are reported in Table 4 and Additional file 3: Table S3. A significantly higher frequency of CIS III was detected in 11 (25.6%) patients injured by the hollow-point bullets as compared to 7 (10.1%) patients injured by shape-stable bullets (p = 0.031). CIS IV was detected in 7 (16.3%) patients injured by the hollow-point bullets as compared to 2 (2.9%) patients injured by shape-stable bullets (p = 0.011).

**Table 4:** Analyses of colon trauma severity by Colon Injury Scale in relation to hollow-point bullets or shape-stable bullets injury

Colon Injury Scale (CIS) parameters		Grouping of patients		All wounded n = 112	p value
		Injured by Shape-stable bullets n = 69	Injured by Hollow-point bullets n = 43		
CIS stage	I	28 (40.6%)	0 (0.0%)	28 (25.0%)	< 0.0001
	II	31 (44.9%)	23 (53.5%)	54 (48.2%)	0.38
	III	7 (10.1%)	11 (25.6%)	18 (16.1%)	0.031
	IV	2 (2.9%)	7 (16.3%)	9 (8.0%)	0.011
	V	1 (1.4%)	2 (4.7%)	3 (2.7%)	0.31

**Fig. 3:**
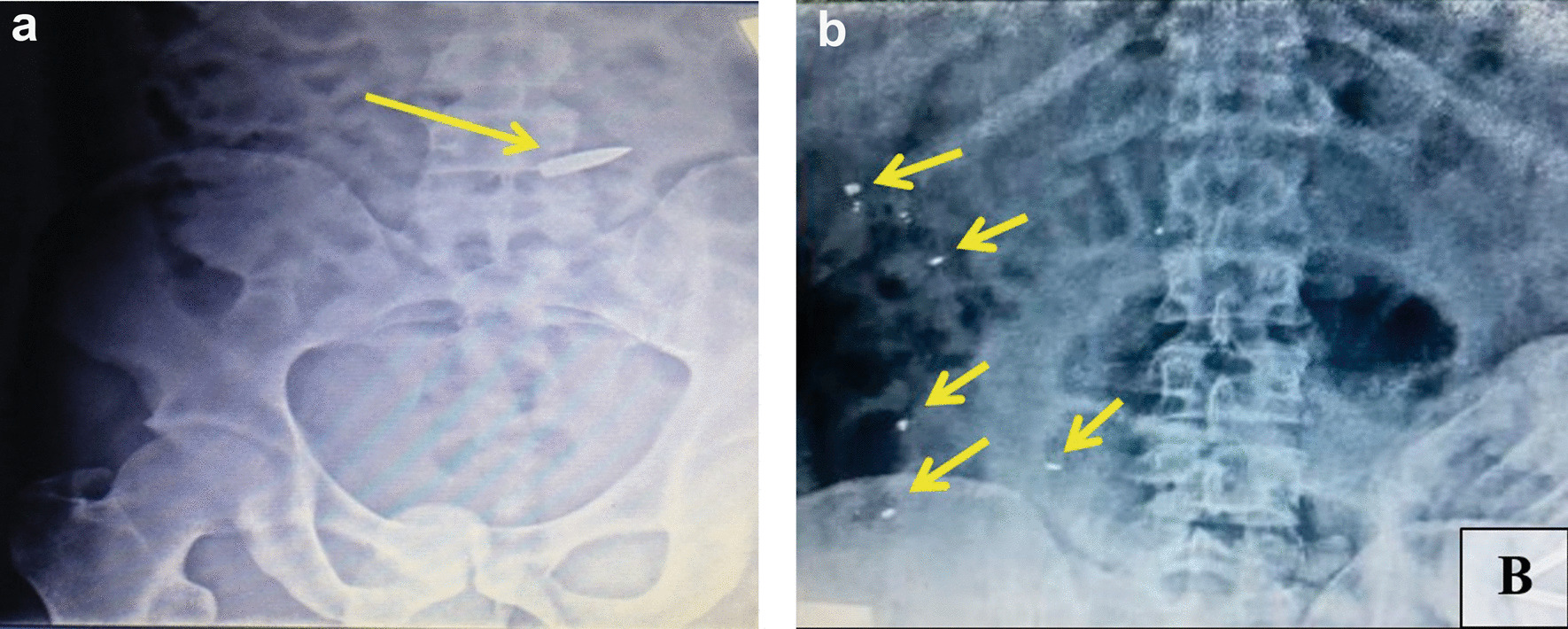
Photographs of X-ray films illustrating intraabdominal fragments with metal density features

**Fig. 4:**
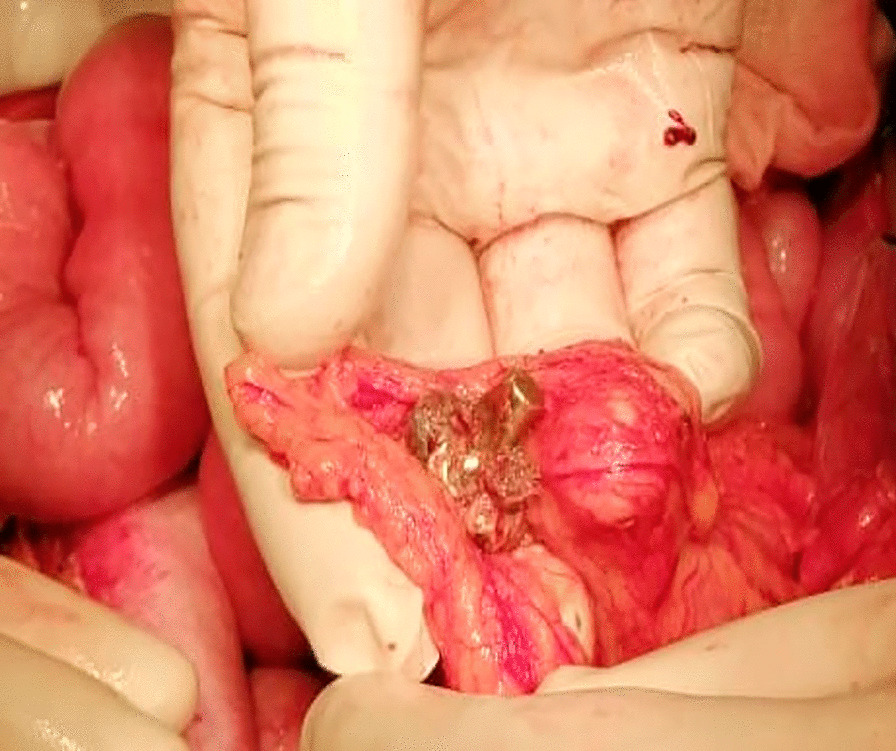
Intraoperative photograph demonstrating hollow-point bullet with mushrooming effect (a yellowish metal object in the arms of the surgeon)

X-ray showed foreign metal density objects in the abdomen in 17 (24.6%) patients injured by shape-stable bullets, which was subsequently confirmed at laparotomy (Fig. 3). In 12 (27.9%) patients injured by hollow-point bullets were found various sizes of multiple bullet fragments in the quantity of up to 5 fragments in 7 (16.3%) patients, 6 to 10 fragments in 13 (30.2%) patients, and 11–15 fragments in 11 (25.6%) patients. Abdomen X-ray examples of bullet fragments are illustrated in Fig. 3. Mushrooming bullets (Fig. 4) were identified in 19 (44.2%) patients injured by hollow-point bullets and in no patients injured by shape-stable bullets (p < 0.0001).

The list of operations is summarized in Table 5. There was no significant difference in the types of operations in the study groups. Colostomy was performed in 14 (20.3%) patients injured by shape-stable bullets and 12 (27.9%) patients injured by hollow-point bullets.

**Table 5:** Analyses of surgical operation in study cohort

Operation type	Injured by shape-stable bullets n = 69	Injured by hollow-point bullets n = 43
Laparoscopic colon wound primary repair, n =	2 (3.0%)	0
Colon wound primary repair, n =	3 (4.3%)	0
Colon wound primary repair + colostomy, n =	0	1 (2.3%)
Colon wound primary repair + small intestine resection, n =	21 (30.4%)	9 (20.9%)
Colon wound primary repair + colostomy + ureter wound primary repair and ureter stenting, n =	0	2 (4.6%)
Colon wound primary repair + cholecystectomy, n =	3 (4.3%)	0
Colon wound primary repair, transversostomy + stomach wound primary repair, n =	4 (5.8%)	2 (4.6%)
Colon wound primary repair + splenectomy, n =	2 (3.0%)	2 (4.6%)
Colon wound primary repair + liver wound primary repair + colostomy, n =	3 (4.3%)	2 (4.6%)
Colon wound primary repair + colostomy + pancreatic wound primary repair, n =	2 (3.0%)	0
Extraperitonization, n =	2 (3.0%)	0
Right hemicolectomy, n =	2 (3.0%)	2 (4.6%)
Right hemicolectomy + ileostomy, n =	0	1 (2.3%)
Right hemicolectomy + right nephrectomy, n =	2 (3.0%)	5 (11.6%)
Right hemicolectomy + liver wound primary repair, n =	14 (20.0%)	5 (11.6%)
Left hemicolectomy, n =	1 (1.4%)	0
Left hemicolectomy + left nephrectomy, n =	3 (4.3%)	8 (18.6%)
Hartmann’s operation, n =	1 (1.4%)	2 (4.6%)
Hartmann’s operation + bladder wound primary repair, n =	4 (5.8%)	2 (4.6%)

Postoperative death was observed in 24 (21.4%) patients. Of these, 15 (35%) patients died after injury by the hollow-point bullet, whereas 9 (13%) patients after damage by the shape-stable bullets (p = 0.0089). Causes of mortality at Level II were massive hemorrhage, heart and respiratory failures. Causes of mortality at Level III were the failure of colorectal anastomoses and anastomotic leak, peritonitis, and perforated peptic ulcer of the small intestine. Causes of mortality at Level IV were sepsis, abdominal abscesses, purulent pneumonia, and acute bowel obstruction.

Detailed follow-up data is unavailable, which is a limitation.

## Discussion

This paper is the first study reporting data from a combat cohort with injury to the colon by hollow-point bullets, which have been used against Ukrainian Armed Forces since 2014. War in Ukraine is associated with specific patterns of colon injuries due to the application of prohibited weapons, including hollow-point pullets, against Ukrainian Armed Forces. Such violation of international war-related law was not described previously in the literature. Although NATO standards are routinely applied for individual body protection by using the tactical helmet and modular tactical vest for the ballistic protection of the neck, chest, abdomen, and groin, our results show severe colon injury. The research is ongoing to develop better individual body protection, considering the damage properties of modified bullets, but details of such research are classified and unavailable. This study highlighted various clinical outcomes of colon injury in case of damage by modern hollow-point bullets as compared to conventional types of shape-stable bullets. This study showed severe trauma and a higher mortality rate for patients injured by hollow-point bullets. Our findings shed light on clinical features and management of gunshot colon injury, which might be helpful in further clinical application for both civil and combat casualties.

Similar to other reports, we showed that most patients were evacuated within the “golden hour” to Level II and treated according to the principle of damage control surgery. Damage control surgery is a standard protocol for Ukrainian military medicine and is in line with other studies of abdominal gunshot trauma [13–16].

Although we have not found publications with a specific reference to the hollow-point bullet injury *vs.* shape-stable bullets, evidence from this study, as well as other experimental studies of internal wound ballistics, confirms our data concerning increased tissue damage by such kind of weapon [8, 9, 17, 18]. Our findings demonstrate that most patients had concurrent damage to other organs, supporting the abovementioned hypothesis.

In line with Busić et al. and Elfaedy et al., we also demonstrated a high incidence of hemorrhagic shock in our patients, suggesting such gunshot complications as a common event [11, 19]. Also, we have shown a higher frequency of hemorrhagic shock in patients injured by hollow-point than shape-stable bullets, which is another evidence of severe trauma from the modern weapon. The higher severity of shock is associated with higher levels of ischemia and necrosis of colon tissues, which act to increase bacterial colonization, resulting in a higher risk for anastomosis leak or failure [11, 20].

Similar to other reports, the left colon part (*i.e.,* splenic colon flexure, descending and sigmoid colon) and middle colon (ascending and descending colon) were frequently injured as compared to other sites [1, 11, 21–24]. The importance to note that colon injury is related to a higher risk of fecal diversion. Watson et al. showed the high risk of fecal diversion in combat patients with left-side colon injury by high-energy projectiles. Our findings also demonstrate a high frequency of left-side colon injury, and we hypothesize that patients with such locations of gunshot wounds have a higher risk for complications and need to undergo a colostomy. However, we also suggest considering the bullet type (*i.e.,* the hollow-point bullet) as an additional factor to increase tissue damage. Our findings would seem to suggest that mechanisms of injury by modern bullets may affect all colon parts without relation to bullet type.

Unlike other research carried out in this area, we showed multiple injuries to the colon in 58% of patients, which is higher compared to other cohorts. For example, Oosthuizen et al. showed 12% and Steele et al. 15% of multiple injuries in patients diagnosed with more than one injury to the colon in the study of non-combat trauma [21, 23]. Cardi et al. demonstrated multiple segment injuries in both large and small bowel in 21.3% of the patients [25]. However, our finding aligns with Bothaigi et al., who showed 64 colorectal wounds in 56 patients in the study of abdominal battlefield trauma [22]. These differences can be explained by the frequent application of the hollow-point bullets in our cohort compared to others.

Analyses of CIS showed that 48.2% of patients had CIS II or greater in the entire cohort. Our observation is consistent with Fealk et al., reporting 59% of patients to have CIS > 2 as well as with Miller et al., who showed a mean CIS of 2.4 [24, 26]. In this study, we showed no patients to have CIS I but a higher frequency of CIS III and IV among individuals injured by the hollow-point bullets compared to shape-stable bullets. These findings could potentially play a significant role in management of the patients injured with expanding bullets as well as provide an evidence of expanding bullets to cause severe colon trauma.

The overall rate of colostomy in our cohort (20.3%) is in contrast to other reports: Steele (33%), Glasgow (36.3%), Watson (36.9%), Oosthuizen et al. (50%) or Mitchao et al. (2.5%) [1, 10, 15, 23, 27]. Cardi et al. showed colostomies in 13.6% of the patients with colorectal wounds in case of high fecal spread or at damage control surgery [25]. As judged from the analyses of other series and our experience, there are multiple factors to play a role in surgeons’ decision to choose a certain operative approach in each situation, including a colostomy. The observed proportion of colostomies could be attributed to severe colon trauma, which is in line with the previously published series [15, 16, 21]. We agree with other researchers suggesting avoiding colostomy and performing primary closure in selected injured patients by shape-stable bullets [15, 16, 21]. However, in this study, colon injury by the hollow-point bullets is associated with more severe trauma, and the decision to perform a colostomy should be made after the evaluation of the abdomen in the context of damage control surgery.

Mortality is usually high in patients with gunshot colon wounds, mainly because of multiorgan injury and severe hemorrhagic shock [15]. In our study, we showed lethal outcomes in 21.4% of patients, which is close to data in studies of Oosthuizen et al., who showed overall mortality of 26%, and Vertrees et al. 29% of patients with gunshot wounds [11, 16]. In contrast, Elfaedy et al. demonstrated mortality of 6.9%; however, in that study, 40% of patients were not in hemorrhagic shock, which is a risk factor for death in patients with an abdominal gunshot injury, including colon [11]. Glasgow et al. showed overall mortality of 9.5% [10]. Steel et al. showed 17.7% mortality in combat patients [23]. Zero colon-related mortality was demonstrated by Bothaigi et al. and Mitchao et al. [22, 27]. Cardi et al. showed a mortality of 12.8%, with hemorrhagic shock as the most frequent cause of death [25]. According to the published series, mortality is reported to be high in multiple gunshot wounds, as also shown in this study [28].

## Conclusions

To sum up, our study demonstrated different colon injury patterns due to gunshot wounds by the hollow-point bullets in the war in Ukraine. These patterns include a higher frequency of hemorrhagic shock (stage II or higher), CIS II or higher, as well as multiple perforations of colon walls. All patients should be suspected to have an injury by bullet with expanding properties in case of penetrating abdominal injury (absent of outlet wound) and careful revision of the abdomen must be performed to identify possible multiorgan injury as well as multiple gunshot defects of the intestine.

## Supplementary Information

**Additional file 1. Table S1.** Analyses of the hemorrhagic shock severity in relation to cause of gunshot injury by shape-stable or hollow-point bullets.

**Additional file 2. Table S2.** Analyses of colon injury by localizations of gunshot defects by shape-stable or hollow-point bullets.

**Additional file 3. Table S3.** Analyses of colon trauma severity by Colon Injury Scale in relation to hollow-point bullets or shape-stable bullets injury.

## Data Availability

All data generated or analyzed during this study are included in this published article.
