# Supplementary material for: Gunshot injury to the colon by expanding bullets in combat patients wounded in hybrid period of the Russian-Ukrainian war during 2014–2020
Source: BMC Surg. 2023 Jan 27;23:23. doi: 10.1186/s12893-023-01919-6 (PMC9883919; doi:10.1186/s12893-023-01919-6)
Supplement: Supplementary file 1 — Additional file 1. Table S1. Analyses of the hemorrhagic shock severity in relation to cause of gunshot injury by shape-stable or hollow-point bullets. [file 12893_2023_1919_MOESM1_ESM.docx]

Additional file 1 (Table S1). Analyses of the hemorrhagic shock severity in relation to cause of gunshot injury by shape-stable or hollow-point bullets

| Shock severity | | Grouping of patients | | All patients  n = 112 | χ^2^-value | P value |
| --- | --- | --- | --- | --- | --- | --- |
|  |  | Injured by Shape-stable bullets  n = 69 | Injured by Hollow-point bullets  n = 43 |  |  |  |
| Stages | I | 25 (36.2%) | 2 (4.7%) | 27 (24.1%) | 14.44 | 0.0001 |
|  | II | 27 (39.1%) | 16 (37.2%) | 43 (38.4%) | 0.04 | 0.8389 |
|  | III | 13 (18.8%) | 18 (41.9%) | 31 (27.7%) | 7.01 | 0.0081 |
|  | IV | 4 (5.8%) | 7 (16.3%) | 11 (9.8%) | 3.29 | 0.0699 |
| χ^2^-test for severity, df = 3 | χ^2^ | 17.57 | 13.45 | 16.14 | n/a | n/a |
|  | p-value | 0.0002 | 0.0012 | 0.0003 |  |  |
| Stages | І-ІІ | 52 (75.4%) | 18 (41.9%) | 70 (62.5%) | 12.69 | 0.0004 |
|  | ІІІ-IV | 17 (24.6%) | 25 (58.1%) | 42 (37.5%) |  |  |
| χ^2^-test for severity, df = 3 | χ^2^ | 31.28 | 2.51 | 9.62 | n/a | n/a |
|  | p-value | <0.0001 | 0.2858 | 0.0082 |  |  |
| Stages | I, IV | 29 (42.0%) | 9 (20.9%) | 38 (33.9%) | 5.26 | 0.0218 |
|  | II-III | 40 (58.0%) | 34 (79.1%) | 74 (66.1%) |  |  |
| χ^2^-test for severity, df = 3 | χ^2^ | 3.37 | 17.78 | 14.62 | n/a | n/a |
|  | p-value | 0.1854 | 0.0001 |  |  |  |
| Descriptive statistic for groups | M | 1.94 | 2.70 | 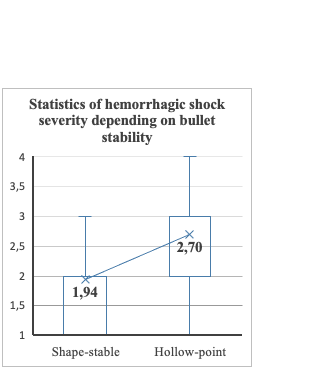  **p < 0.0001** | | |
|  | df | 68 | 42 |  |  |  |
|  | SD | 0.89 | 0.80 |  |  |  |
|  | SD^2^ | 0.79 | 0.64 |  |  |  |
|  | V_R_ | 154.5% | 111.2% |  |  |  |
|  | V_d_ | 35.2% | 25.1% |  |  |  |
|  | V_σ_ | 45.8% | 29.8% |  |  |  |
|  | CQD | 0.33 | 0.20 |  |  |  |
|  | Mo | 2 | 3 |  |  |  |
|  | Me | 2 | 3 |  |  |  |
|  | IQR | 1 | 1 |  |  |  |
| Student t-test | T | 0.63 | |  |  |  |
|  | p-value | 0.53 | |  |  |  |

Table notes: χ^2^ – value of inverse probability of χ^2^ distribution; p-value – probability; M – AMV, arithmetic mean value; df – degree of freedom; SD – standard deviation; SD^2^ – variance; V_R_ – relative range; V_d_ – RMD, relative mean difference; V_σ_ – CV, coefficient of variation; CQD - coefficient of quartile deviation; Me – median; Mo – mode; IQR – interquartile range; T – inverse value of the cumulative t-distribution
