# Supplementary material for: Gunshot injury to the colon by expanding bullets in combat patients wounded in hybrid period of the Russian-Ukrainian war during 2014–2020
Source: BMC Surg. 2023 Jan 27;23:23. doi: 10.1186/s12893-023-01919-6 (PMC9883919; doi:10.1186/s12893-023-01919-6)
Supplement: Supplementary file 2 — Additional file 2. Table S2. Analyses of colon injury by localizations of gunshot defects by shape-stable or hollow-point bullets. [file 12893_2023_1919_MOESM2_ESM.docx]

Additional file 2 (Table S2). Analyses of colon injury by localizations of gunshot defects by shape-stable or hollow-point bullets

| Colon parameters | | Grouping of patients | | All patients  n = 112 | χ^2^-value | p value |
| --- | --- | --- | --- | --- | --- | --- |
|  |  | Injured by Shape-stable bullets  n = 69 | Injured by Hollow-point bullets  n = 43 |  |  |  |
|  |  |  |  |  |  |  |
| Colon part | Cecum | 6 (8.7%) | 2 (4.7%) | 8 (7.1%) | 0.65 | 0.42 |
|  | Ascending colon | 9 (13.0%) | 9 (20.9%) | 18 (16.1%) | 1.22 | 0.27 |
|  | Hepatic flexure | 3 (4.3%) | 4 (9.3%) | 7 (6.3%) | 1.11 | 0.29 |
|  | Transverse colon | 10 (14.5%) | 7 (16.3%) | 17 (15.2%) | 0.07 | 0.80 |
|  | Splenic flexure | 5 (7.2%) | 1 (2.3%) | 6 (5.4%) | 1.27 | 0.26 |
|  | Descending colon | 21 (30.4%) | 12 (27.9%) | 33 (29.5%) | 0.08 | 0.78 |
|  | Sigmoid colon | 15 (21.7%) | 8 (18.6%) | 23 (20.5%) | 0.16 | 0.69 |
| χ^2^-test for segment, df = 6 | χ^2^ | 24.03 | 15.44 | 36.75 | n/a | n/a |
|  | p-value | 0.0005 | 0.0171 | < 0.0001 |  |  |
| *χ^2^-test for segment, df = 6 | χ^2^ | 7.59 | 3.63 | 7.55 |  |  |
|  | p-value | 0.27 | 0.73 | 0.27 |  |  |
| Colon side | Right-side colon | 18 (26.1%) | 15 (34.9%) | 33 (29.5%) | 0.99 | 0.32 |
|  | Transverse colon | 10 (14.5%) | 7 (16.3%) | 17 (15.2%) | 0.07 | 0.80 |
|  | Left-side colon | 41 (59.4%) | 21 (48.8%) | 62 (55.4%) | 1.20 | 0.27 |
| χ^2^-test for side, df = 2 | χ^2^ | 22.52 | 6.88 | 27.88 | n/a | n/a |
|  | p-value | < 0.0001 | 0.032 | < 0.0001 |  |  |
| Colon level | upper colon | 18 (26.1%) | 12 (27.9%) | 30 (26.8%) | 0.04 | 0.83 |
|  | middle colon | 30 (43.5%) | 21 (48.8%) | 51 (45.5%) | 0.31 | 0.58 |
|  | lower colon | 21 (30.4%) | 10 (23.3%) | 31 (27.7%) | 0.68 | 0.41 |
| χ^2^-test for level df = 2 | χ^2^ | 3.39 | 4.79 | 7.52 | n/a | n/a |
|  | p-value | 0.18 | 0.091 | 0.023 |  |  |
| Relation to peritoneum | intraperitoneal | 39 (56.5%) | 22 (51.2%) | 61 (54.5%) | 0.31 | 0.58 |
|  | extraperitoneal | 30 (43.5%) | 21 (48.8%) | 51 (45.5%) |  |  |
| χ^2^-test for level, df = 1 | χ^2^ | 0.59 | 0.01 | 0.45 | n/a | n/a |
|  | p-value | 0.44 | 0.91 | 0.50 |  |  |
| Typical parts / other parts | **typical parts | 50 (72.5%) | 30 (69.8%) | 80 (71.4%) | 0.09 | 0.76 |
|  | other parts | 19 (27.5%) | 13 (30.2%) | 32 (28.6%) |  |  |
| χ^2^-test for typical parts, df = 1 | χ^2^ | 7.33 | 3.50 | 10.78 | n/a | n/a |
|  | p-value | 0.0068 | 0.061 | 0.0010 |  |  |

Table notes: χ^2^ – value of inverse probability of χ^2^ distribution; p-value – probability; df – degree of freedom; * – χ^2^-test calculated with correction for length of colon segment: length of ascend. transverse. descend & sigmoid colon is conditionally treated as three times longer than length of cecum. hepatic & splenic flexure; ** – typical segments are considered as parts of colon. where statistical analysis was shown the highest frequency of gunshot injury: sigmoid. ascending & descending colon. splenic flexure; n/a – not applicable
