# Supplementary material for: Gunshot injury to the colon by expanding bullets in combat patients wounded in hybrid period of the Russian-Ukrainian war during 2014–2020
Source: BMC Surg. 2023 Jan 27;23:23. doi: 10.1186/s12893-023-01919-6 (PMC9883919; doi:10.1186/s12893-023-01919-6)
Supplement: Supplementary file 3 — Additional file 3. Table S3. Analyses of colon trauma severity by Colon Injury Scale in relation to hollow-point bullets or shape-stable bullets injury. [file 12893_2023_1919_MOESM3_ESM.docx]

Additional file 3 (Table S3). Analyses of colon trauma severity by Colon Injury Scale in relation to hollow-point bullets or shape-stable bullets injury

| Colon Injury Scale (CIS) parameters | | Bullet type groups | | All wounded  n = 112 | χ^2^-value | P value |  |
| --- | --- | --- | --- | --- | --- | --- | --- |
|  |  | Injured by Shape-stable bullets  n = 69 | Injured by Hollow-point bullets  n = 43 |  |  |  |  |
| CIS stage | I | 28 (40.6%) | 0 (0.0%) | 28 (25.0%) | 23.27 | <0.0001 |  |
|  | II | 31 (44.9%) | 23 (53.5%) | 54 (48.2%) | 0.78 | 0.38 |  |
|  | III | 7 (10.1%) | 11 (25.6%) | 18 (16.1%) | 4.68 | 0.031 |  |
|  | IV | 2 (2.9%) | 7 (16.3%) | 9 (8.0%) | 6.42 | 0.011 |  |
|  | V | 1 (1.4%) | 2 (4.7%) | 3 (2.7%) | 1.04 | 0.31 |  |
| χ^2^-test for CIS, df = 4 | χ^2^ | 54.45 | 32.72 | 64.45 | n/a | n/a |  |
|  | p-value | <0.0001 | <0.0001 | 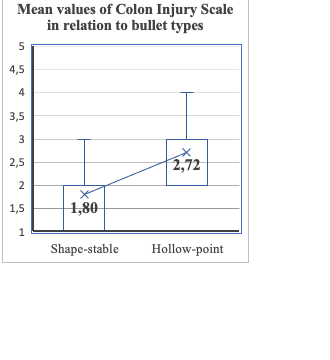<0.0001 |  |  |  |
| Descriptive statistic for groups | M | 1.80 | 2.72 | \|  \| \| --- \| | | | |
|  | SD | 0.85 | 0.91 |  |  |  |  |
|  | SD^2^ | 0.72 | 0.83 |  |  |  |  |
|  | SEM | 0.10 | 0.14 |  |  |  |  |
|  | R | 4 | 3 |  |  |  |  |
|  | V_R_ | 222.6% | 110.3% |  |  |  |  |
|  | V_d_ | 36.0% | 28.3% |  |  |  |  |
|  | V_σ_ | 47.3% | 33.4% |  |  |  |  |
|  | CQD | 0.33 | 0.20 |  |  |  |  |
|  | Mo | 2 | 2 |  |  |  |  |
|  | Me | 2 | 2 |  |  |  |  |
|  | IQR | 1 | 1 |  |  |  |  |
| One-way ANOVA | F_(1, 110)_ | 29.67 | |  |  |  |  |
|  | p-value | <0.0001 | |  |  |  |  |

Table notes: ANOVA – Analysis of Variance; R – range of the sample; SEM – standard error of mean value; F – inverse value of the cumulative F-distribution; statistical values – see endnotes for table 1, n/a – not applicable
